# Editorial: Lactic Acid Bacteria Within the Food Industry: What Is New on Their Technological and Functional Role

**DOI:** 10.3389/fmicb.2021.711013

**Published:** 2021-07-09

**Authors:** Paola Lavermicocca, Cristina Reguant, Joaquin Bautista-Gallego

**Affiliations:** ^1^Institute of Sciences of Food Production, National Council of Research of Italy, Bari, Italy; ^2^Departament de Bioquímica i Biotecnologia, Facultat d'Enologia, Universitat Rovira i Virgili, Tarragona, Spain; ^3^Departamento de Ciencias Biomédicas (Área de Microbiología), Facultad de Ciencias, Universidad de Extremadura, Badajoz, Spain

**Keywords:** fermentation processes, product preservation, food quality, food safety, health promoting features

## Introduction

The application of lactic acid bacteria (LAB) in food processing has a very long history (Leroy and De Vuyst, 2004), however the research continuously provides new insight. The biotransformation performed by LAB is implemented in many industrial processes since selected pro-technological LAB—through their lactic acid fermentative activity—can start and/or modulate the fermentation by producing acidic metabolites and other biomolecules such as enzymes, antimicrobials, and molecules contributing to aroma and texture. Through their proteolytic activity, LAB are able to degrade proteins into small peptides and free amino acids converted, in turn, to various metabolites—alcohols, aldehydes, acids, and ester compounds. Furthermore, lipolysis, glycolysis, and pyruvate metabolism performed by LAB produce many metabolites including aromatic compounds acting as flavor compounds (Leroy and De Vuyst, 2004). All these molecules contribute to the overall quality, shelf-life, and safety of foods, by enhancing their technological, sensorial, nutritional and functional features (Ravyts et al., 2012). It is worthy to note that the functional relevance of biotransformation operated by LAB relies also to their ability in modifying the bioavailability of bioactive molecules composing the food (Debelo et al., 2020). To successfully manage food processing and provide consumers with healthy foods, the genetic traits, and metabolisms of LAB have been deeply investigated in order to select strains suitable for specific applications (Giraffa, 2014).

The most common food-related LAB include species of the genera *Lactobacillus, Lactococcus, Leuconostoc, Enterococcus, Pediococcus, Streptococcus*, and *Weissella*. Within the phylum *Firmicutes*, the majority of LAB belongs to the order of *Lactobacillales*. Recently, the genus *Lactobacillus* has been reclassified into 25 genera (Zheng et al., 2020). However, to be applied in food processing LAB species must be recognized as safe (GRAS) by Food and Drug Administration (FDA) or must have achieved the Qualified Presumption of Safety (QPS) status by European Food Safety Authority (EFSA) (EFSA Panel on Biological Hazards et al., 2017).

Foods represent also the main source of LAB strains having probiotic features. Besides, food components play a role as vector in delivering probiotic populations by protecting them through the gastro-intestinal tract and by sustaining their gut colonization (Flach et al., 2018). Probiotic bacteria—defined as “live microorganisms that, when administered in adequate amounts, confer a health benefit on the host” (Hill et al., 2014)—mainly belonging to *Lactobacillus* and *Bifidobacterium* genera, are widely used in commercial products, particularly in milk-based preparations, that fall in the category of functional foods. Gut colonization by individual strains selected for beneficial functions strengthen the barrier function played by the epithelial and endothelial gut cells. Furthermore, their implantation modifies the resident microbial populations favoring the increase of those beneficial while limiting potential pathogens and, by modulating the components of the intestinal immune system, may stimulate the host immune response (Chugh and Kamal-Eldin, 2020). In the last decades, consumer's and market's interest for foods characterized by functional attributes has determined the widespread of research on probiotic foods. Particular interest is devoted to vegetable and fruit matrices rich in bioactive molecules that may act as carrier for probiotic microorganisms, producing innovative symbiotic (Flach et al., 2018).

Furthermore, the research and commercial interest in selecting strains for specific food processing, suggest the need for an update on the ongoing studies regarding metabolisms, genetic traits, fermentation performances, and functional features of LAB. Thus, the information obtained in this Research Topic can provide the food industry with rigorous scientific demonstrations for even more efficient biotransformation processes.

The Research Topic “Lactic Acid Bacteria within the Food Industry: What is New on their Technological and Functional Role” belongs to the Food Microbiology section in the Frontiers in Microbiology journal. It covers a total of 12 contributions divided in two reviews and 10 original research papers. Many of the most relevant researchers in the field have collaborated in its elaboration.

We present an overview of these papers which can be grouped under different research themes as follows: (i) probiotic and health-promoting characteristics for the production of functional foods and beverages; (ii) Interactions with other microorganisms; (iii) new technological approaches. The diversity of research displayed in this Research Topic demonstrates the important potential of these microorganisms and its impact in the food industry.

In the first group of papers, Hernández-Alcántara et al. have evaluated different probiotic properties of *L. plantarum* strains, such as their survival in the human digestive tract and *in vivo* studies using a murine model. Two strains (M5MA1-B2 and M9MG6-B2) were able to tolerate the gastrointestinal stresses and displayed *in vitro* high adhesion capacity to Caco-2 cells. Furthermore, they showed great biofilm formation characteristics which indicate a potential capability for intestine colonization, and an improvement of the surface area of the intestinal epithelium in the *in vivo* murine model. A similar study was carried out by Baliyan et al. who isolated potential probiotics microorganisms from a cereal-based traditional fermented beverage -lugri- (rice, wheat and barley) focusing in the functional and safety characteristics of this kind of beverage. *Lacticaseibacillus paracasei* LUL:01 exhibited the best performance and potential for its application in functional food formulation (milk-based formulation).

In addition, Cordeiro et al. have described the therapeutic effect in an induced colitis mice model of a Minas Frescal cheese made using the probiotic bacteria *L. lactis* NCDO 2118. They showed that this microorganism was able to limit the histopathological damages and restore intestinal barrier by increasing expression of gene related to tight junction protein and modulated cytokine production in mice. Furthermore, this functional cheese was able to produce high levels of bioactive peptides with antihypertensive, antioxidant, and antidiabetic activities.

Verni et al. studied the use of brewers' spent grain, a by-product of the brewing industry, as a source to increase the intake of antioxidant compounds. To enable the release or synthesis of these compounds, a fermentation with different selected *Lactiplantibacillus plantarum* strains and treatment with a commercial xylanase was carried out.

Cataldo et al. evaluated the production of gamma-aminobutyric acid through fermentation of strawberry and blueberry juices by *Levilactobacillus brevis* CRL 2013. A significantly higher production was reached in the case of fermented strawberry juice and it was able to modulate the expression of *cox-2* in lipopolysaccharide stimulated macrophages and exerted a remarkable anti-inflammatory effect. This study supports the potential use of this kind of fermented juice to reduce the inflammatory response of chronic inflammatory diseases.

Regarding to the interactions of LAB with other microorganisms, Medved'ová et al. have studied the effect of different combinations of LAB on the growth of different *Staphylococcus aureus* and *Escherichia coli* strains during the ripening of curd cheeses. This study emphasizes the importance of the use of LAB starter cultures to improve the sensory profile and safety conditions of the final product. In the same trend, Canon et al. reviewed the different ways available at present that could be used to create positive interactions between LAB: different types of positive interactions; co-cultivation and their mechanisms to reach the positive interactions; possible strategies that could be used to assemble LAB; and the particular role of nutritional dependencies.

With concern of the new technological approaches for LAB, four original articles and one review overview different studies to their implementation. Speranza et al. have studied the optimization of a fish fermented product using two *L. plantarum* strains. Both strains reduced the fermentation time and ensured good microbiological, chemico-physical, and sensorial quality of the final product.

Liu et al. have provided an approach to investigate the molecular mechanisms of formation and metabolic pathways of flavors in rice-acid fermented with *L. paracasei* H4-11 at different time and inoculation (co-inoculation with *Kluyveromyces marxianus*) methods. At transcriptional level, they detected that the genes related to amino sugar and nucleotide sugar metabolism and starch and sucrose metabolism affected the energy required for the growth of *L. paracasei* in the early stage. Even more, a different expression of those genes was detected in the growth of *L. paracasei* in the presence of *K. marxianus*.

Valerio et al. delved in the selection of LAB strains able to produce EPS in liquid sourdoughs based on pseudocereal flours and evaluated the effect of its composition on EPS production and protein degradation. Thus, the modulation of flour type, DY and sucrose content can stimulate the metabolic activities of *Weisella cibaria* and *L. plantarum* modulate the fermentation process, enriched in the EPS content. Furthermore, this increment in the EPS production was detected in the presence of pseudocereals amaranth or quinoa.

Kazou et al. compared the bacterial (and yeast/fungal) microbiota of different Greek kefir samples, using classical microbiological and amplicon-based metagenomics approaches. They identified mainly *Lentilactobacillus kefiri, Leuconostoc mesenteroides, Lacticaseibacillus rhamnosus, Streptococcus thermophilus, Lactococcus lactis*, and *Leuconostoc mesenteroides*. Yeasts were also isolated and identified during kefir production, being *K. marxianus, Debaryomyces hansenii*, and *Saccharomyces cerevisiae* the mostly identified yeast species. Even more, some (opportunistic) pathogens were detected at home-made kefir samples, which indicates poor hygiene practices.

Finally, Virdis et al. have reviewed the role of lactic acid bacteria in wine, focusing on the malolactic fermentation. Furthermore, they delved in the study of: (i) their contribution to the sensorial profile (citrate and glycosidase metabolism); (ii) impact on wine color (reduction of anthocyanin glucosides); (iii) production of volatile thiols; (iv) other diverse activities (reduction of the use of bentonite); and their negative effects (biogenic amines, ethyl carbamate).

The varied contributions to this Research Topic are evidence of the study undertaken by researchers that provide an updated and high-quality overview of the impact of lactic acid bacteria and their future perspectives. We hope that this Research Topic informs readers properly about the benefit of this product and the challenges that have yet to be overcome in this field.

## Author Contributions

All authors listed have made a substantial, direct and intellectual contribution to the work, and approved it for publication.

## Conflict of Interest

The authors declare that the research was conducted in the absence of any commercial or financial relationships that could be construed as a potential conflict of interest.
